# Dynamin 3 Inhibits the Proliferation of Non-small-Cell Lung Cancer Cells by Suppressing c-MET–GBR2–STAT3 Complex Formation

**DOI:** 10.3389/fcell.2021.641403

**Published:** 2021-08-19

**Authors:** Qiang Lu, Yunfeng Ni, Wuping Wang, Lei Wang, Tao Jiang, Lei Shang

**Affiliations:** ^1^Department of Thoracic Surgery, Tangdu Hospital, The Air Force Military Medical University, Xi’an, China; ^2^The Ministry of Education Key Lab of Hazard Assessment and Control in Special Operational Environment, Department of Health Statistics, School of Public Health, The Air Force Military Medical University, Xi’an, China

**Keywords:** dynamin 3, lung cancer, non-small-cell lung cancer, growth factor receptor-bound protein 2, antitumor effect

## Abstract

Dynamin 3 (DNM3) has gained increased attention ever since its potential as a tumor suppressor was reported. However, its action in lung cancer (LC) is undefined. In this study, the role of DNM3 in LC development was investigated. DNM3 expression was found to be downregulated in tumors of patients with LC, especially those with metastasis. The DNM3 downregulation enhanced the proliferative and metastatic ability of LC cells, whereas its upregulation had the opposite effects. *In vivo* xenograft experiments confirmed that lung tumors with lower DNM3 expression had higher growth and metastatic abilities. Mechanistic studies revealed that DNM3 interacts with growth factor receptor-bound protein 2 (GBR2), thereby interrupting tyrosine-protein kinase Met (c-MET)–GBR2–signal transducer and activator of transcription 3 (STAT3) complex formation, which suppressed STAT3 activation. Therefore, the absence of DNM3 frees GBR2 to activate STAT3, which regulates the expression of genes related to LC proliferation and metastasis (e.g., cyclin D1 and Snail family transcriptional repressor 1). Additionally, the c-MET inhibitor crizotinib effectively suppressed LC cell proliferation and migration *in vitro* and *in vivo*, even with DNM3 depleted. Therefore, our study has demonstrated the antitumor effect of DNM3 in LC and suggests that the inhibition of c-MET might be a promising strategy for treating those LC patients with low DNM3 expression.

## Introduction

Lung cancer (LC) is a common malignancy worldwide with a high mortality rate, accounting for 27% of cancer−related deaths in the United States in 2018 (Bray et al., 2018; Siegel et al., 2018). Although the significant progress made in the treatment of non-small-cell lung cancer (NSCLC) through individualized therapy and immunotherapy is very effective in some patients, the 5−year overall survival (OS) of patients with different stages of NSCLC is still only 18%, and that of patients with metastatic tumor is only 5% (Siegel et al., 2018). With the increasing environmental pollution in China, the LC-related morbidity and mortality rates have reached the highest levels in the world (Chen et al., 2016). In recent years, increasing research has been devoted to investigating the use of molecular predictors for prognosis in cancer patients, which can also be used as specific therapeutic targets. Because of the high incidence and mortality rate of LC, there is an urgent need to identify the key molecular predictors of LC pathogenesis.

Cancer is associated with increased cell proliferation and migration, resulting in aggressive cell invasion and metastatic disorders. Dynamins (DNMs) are a family of guanylate triphosphatases (GTPases) that participate in vesicle budding and membrane severing via the hydrolysis of GTPs (Heymann and Hinshaw, 2009). DNM3 is a member of the DNM family that is essential for endocytosis and possesses mechanochemical properties important for actin-membrane processes (Hinshaw, 2000). The role of DNM3 in malignancies had remained unknown until Shen et al. (2012) showed that its promoter was hypermethylated in hepatocellular carcinoma (HCC). Subsequently, Inokawa et al. (2013) found that *DNM3* might be an anti-HCC gene candidate. Furthermore, DNM3 was reported as a tumor suppressor in papillary thyroid carcinoma (Lin et al., 2019), colon cancer (Ma et al., 2019), and breast cancer, and other types of carcinoma (Uehiro et al., 2016). However, the activity of DNM3 in LC is still not yet understood, and its precise function as a tumor suppressor remains unclear.

Therefore, this study aimed to assess the antitumor effects of DNM3 in LC and explore its potential tumor suppression mechanisms. We found that DNM3 expression was abnormally low in the LC tissue and correlated to poor patient survival. The experimental knockdown of *DNM3* using short hairpin RNA (shRNA) promoted the proliferative and metastatic capacities of the LC cells. As to the mechanism involved, the absence of DNM3 enhanced the interaction among growth factor receptor-bound protein 2 (GRB2), tyrosine-protein kinase Met (c-MET), and signal transducer and activator of transcription 3 (STAT3), resulting in STAT3 activation. The depletion or inhibition of c-MET could suppress the tumor growth and metastasis caused by the low expression of DNM3. Our results indicated that the c-MET inhibitor, crizotinib, could be used as a target therapy drug to treat those LC patients with low DNM3 expression.

## Materials and Methods

### Reagents, Cell Lines, and Culture Conditions

The primary anti-DNM3 antibody was purchased from Abcam (Cambridge, United Kingdom). The primary anti-Snail family transcriptional repressor 1 (SNAI1) antibody (SC-113766) was purchased from Santa Cruz (Dallas, United States). The primary anti-GRB2 antibody (#36344), anti-c-MET antibody (#3127), anti-STAT3 antibody (#9139), anti-p-STAT3 antibody (#9145), anti-cyclin D1 (CCND1) antibody (#2978), and anti-glyceraldehyde 3-phosphate dehydrogenase (GAPDH) antibody (#2118) were purchased from Cell Signaling Technology (Danvers, United States). The mouse (SC-2004) and rabbit (SC-2005) source second antibodies for the western blot were purchased from Santa Cruz.

The non-cancerous pulmonary epithelial cell line (BEAS-2B) and LC cell lines (A549, H460, H1299, Calu-3, and H1838) were purchased from the Cell Bank of Type Culture Collection of the Chinese Academy of Sciences (Shanghai, China). The cell lines were cultured in RPMI 1640 medium (Gibco, Carlsbad, United States) supplemented with 10% fetal bovine serum (Gibco) and 100 IU/mL penicillin or 100 mg/mL streptomycin (Gibco) and stored in 37°C incubators under 5% carbon dioxide. For the experiment, the cells were seeded in 12-wells plates or 96-well plates at 40∼50% confluence.

### Patient Specimens

Intraoperatively obtained cancerous and adjacent non-cancerous lung tissue specimens from 51 LC patients, who had been admitted to the Department of General Surgery of The Air Force Military Medical University (Xi’an, China) from January 2014 to August 2019, were used in this study. The patients comprised 31 men and 20 women in the age range of 59–79 years (average age, 64.3 years). All the patients were diagnosed with primary LC through pathological examination, and 23 patients were found to have cancer metastasis. This study was approved by the Medical Ethics Committee of The Air Force Military Medical University (Xi’an, China), and all patients signed consent forms before their participation in the research.

### Animal Experiments

According to national and international guidelines, the animal study was performed, and the protocol was approved by the Institutional Animal Care and Use Committee of The Air Force Military Medical University (Xi’an, China). The female nude mice (4 weeks of age, weighing 15–18 g) were purchased from the Shanghai Institute of Materia Medica (Shanghai, China). For the tumor growth study, mice were injected subcutaneously (4 × 10^6^ cells) with either control shRNA transfected H1299 cells as the control group or *DNM3*-knockdown H1299 cells. Ten days after the tumor inoculation, the tumor size and body weight were monitored, and the mice were then oral gavage with either 10% ethanol in PBS (Control) or 35 mg/kg crizotinib (CZT; 35 mg/kg in 10% ethanol in PBS; Sigma-Aldrich, St. Louis, MO, United States), once a day for 12 days. After administering these solutions, the tumor progress was monitored every 3 days, and all mice were euthanized on the 28th day.

For the observation of lung tumor metastasis, 6-week-old female nude mice were injected with 1 × 10^6^/100 μL H1299 cells (with or without stable *DNM3* knockdown) into the tail veins (5 mice in each group). At 3 weeks after the cell injection, all the animals were euthanized with carbon dioxide, and the lung tissues were extracted. The tumor metastasis in the lungs was observed by counting the tumor nodules under a dissecting microscope.

### Total RNA Extraction and Quantitative Reverse-Transcription Polymerase Chain Reaction

RNA was extracted from the patient tissue samples and cell lines using the TRIzol^®^ reagent and then reverse transcribed into cDNA using the PrimeScript^TM^ RT Master Mix. The RNA quality was monitored by A260 spectrophotometry. The relative mRNA level was measured with the quantitative polymerase chain reaction (qPCR) using an SYBR^®^ Premix Ex Taq^TM^ (Tli RNaseH Plus) PCR kit and an Applied Biosystems Prism^®^ 7300 sequence detector. The relative mRNA expression level was determined with the 2^–ΔΔCq^ method (Livak and Schmittgen, 2001). The GAPDH or β-ACTIN was used as reference genes (Saviozzi et al., 2006). The primer sequences (5′–3′) used were as follows: DNM3 forward, TCGAGGGTCGGGCATTGTA; DNM3 reverse, CTTCAATCTCAAGGCGAACTTCA; CCND1 forward, CCCTCGGTGTCCTACTTCAAA; CCND1 reverse, CCAGGTTCCACTTGAGCTTGT; SNAI1 forward, AATCG GAAGCCTAACTACAGCG; SNAI1 reverse, GTCCCAGATG AGCATTGGCA; GAPDH forward, TGTGGGCATCAATGGA TTTGG; and GAPDH reverse, ACACCATGTATTCCGGGT CAAT; β-ACTIN forward, GACCTGACAGACTACCTCAT; β-ACTIN reverse, AGACAGCACTGTGTTGGCTA.

### Western Blot Analysis

All protein extractions were carried out with NP40 extraction buffer. Western blotting was carried out according to a previous protocol (Niu et al., 2019). The proteins were first separated by electrophoresis, and the protein bands were then electroblotted onto polyvinylidene fluoride (PVDF) membranes. The membranes were incubated with the primary antibodies to the target proteins (all diluted 1:500, except for anti-GADPH, which was diluted 1:1,000). After washing with TBST (Tris-Buffered Saline, 0.1% Tween 20 Detergent), the PVDF membranes were incubated with secondary antibodies. After washing with TBST, the signal development was carried out with enhanced CL systems (ZSGB-Bio, Beijing, China), and signal visualization was carried out with CL Imaging Systems (Tanon 5200) (Tanon Science and Technology, Shanghai, China).

### Cell Growth and 5-Bromo-2′-Deoxyuridine Tests

For the cell growth test at different time points, the MTS assay was carried out with an MTS test kit (Thermo Fisher Scientific). A Wallac Victor 1420 Multilabel Counter was used to measure the absorbance. Each test was repeated 3 times. For the 5-bromo-2′-deoxyuridine (BrdU) test, cells were cultured for 3 days and then labeled with BrdU (3 μg/mL) (Thermo Fisher Scientific) for 4 h. The BrdU results were analyzed according to a previously described method (DeWaal et al., 2018).

### Cell Migration Test

The metastatic capacity of the cells was evaluated by carrying out Transwell migration assays in 24-well culture plates as described by the manufacturer guidelines (Thermo Fisher Scientific). After removing cells at the top of the membrane, the migrated cells were dyed with crystal violet (Thermo Fisher Scientific). Five fields (magnification 40×) were chosen randomly to count the number of migrated cells under an optical microscope (Zeiss Axio Observer, Zeiss, Oberkochen, Germany).

### Immunoprecipitation and Proximity Ligation Assay

Cells were harvested and sonicated with 1 mL of lysis buffer (50 mmol/L Tris-HCl, pH 7.5, 100 mmol/L NaCl, 0.5% Non-idet P-40) supplemented with a protease inhibitor cocktail (Roche Applied Sciences). After centrifugation at 10,000 × *g* for 10 min, the supernatants were load on protein G/A-agarose beads (Invitrogen) with 1–2 μg of antibodies for immunoprecipitation (IP). After incubation for 6 h at 4°C, the beads were washed thrice with PBS (pH 7.4). Beads were then boiled in 2 × Laemmli buffer and subjected to SDS-PAGE and Western blotting.

The proximity ligation assay (PLA) for the interaction of STAT3 and c-MET was conducted using the PLA kit (Sigma-Aldrich) as described by the manufacturer.

### Immunofluorescence and Immunochemical Staining

For immunofluorescence, cells in chamber slides were stained with anti-STAT3 (Cell Signaling) overnight at 4°C, following by secondary staining with the anti-rabbit Alexa Fluor 488–conjugated secondary antibody (Invitrogen) for 1 h at room temperature. Images were taken by fluorescence microscope (Olympus Imaging America, Inc.). The immunochemical staining of Ki-67 was performed on 5-mm paraffin-embedded tumor sections as previously described (Cina et al., 1997).

### Lentivirus and Small Interfering Ribonucleic Acid Transfection

Human *DNM3* shRNA and a scrambled shRNA control and pLenti-*DNM3* cDNA (GenePharma, Shanghai, China) were, respectively, transfected into HEK-293T human embryonic kidney cells with a package of plasmids (VSVG, REV, and pMDL; Addgene, Cambridge, MA, United States) using Lipofectamine 3000 (Thermo Fisher Scientific). After 36 h, targeted cell transfection of the A549 and H460 cell lines was performed using 8 μg/mL polybrene and the virus-containing supernate from the infected HEK-293T culture. The targeted cell transfection of small interfering ribonucleic acid (siRNA) for *c-MET*, *STAT3*, and *GBR2* (Santa Cruz Biotechnology) was performed using Lipofectamine 3000, as described by the manufacturer’s protocol.

### Statistical Analysis

The result was showed with Mean (the mean value for 3 independent replicates) ± SD. The differences between groups were analyzed by Student’s *t*-test or One-Way ANOVA, with *P* < 0.05 indicating statistical significance. Each experiment was repeated 3 times.

## Results

### Low *DNM3* Expression in Lung Tumors Is Related to Low Patient Survival

**FIGURE 1:**
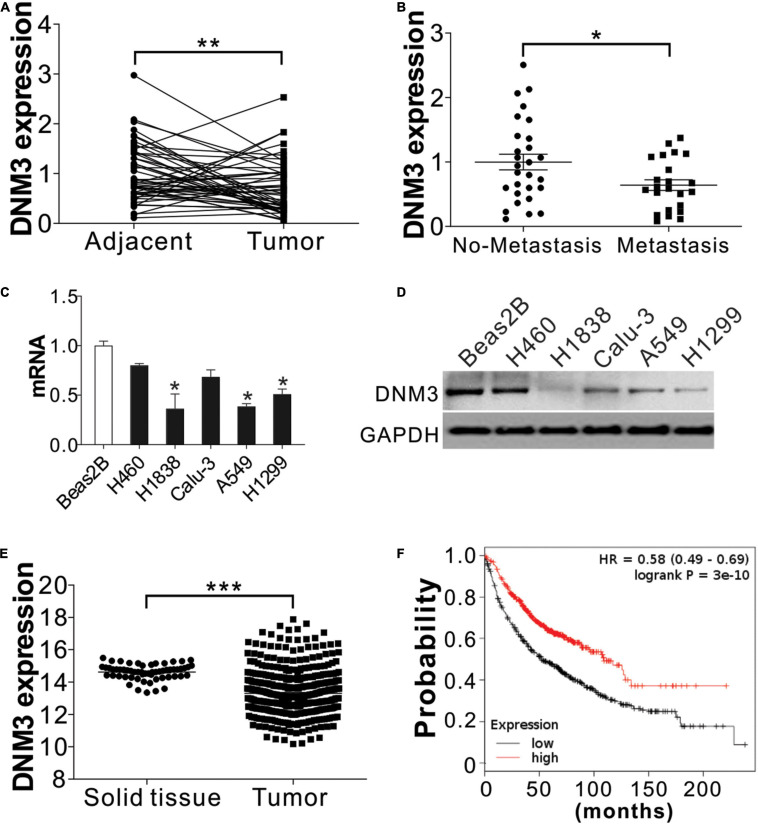
DNM3 expression is low in LC cells *in vivo* and *in vitro*. **(A)** mRNA level of *DNM3* in 51 pairs of adjacent tissues and primary tumors from patients with lung cancer (LC). β-ACTIN was used as reference gene. **(B)** mRNA level of *DNM3* in the tumor samples from patients with (*n* = 23) or without (*n* = 28) cancer metastasis. β-ACTIN was used as the reference gene. **(C)** mRNA level of *DNM3* in the indicated cells. GAPDH was used as the reference gene. **(D)** The protein level of DNM3 in the indicated cells. **(E)** Expression of DNM3 in LC tumors and solid normal tissues according to database of cancer browser (https://xena.ucsc.edu/welcome-to-ucsc-xena/). **(F)** Survival of patients with high- or low-DNM3-expressing LC based on data analysis with the Kaplan–Meier plotter (http://kmplot.com/analysis/index.php?p=service&cancer=lung). Experiments in **(C,D)** were repeated 3 times. *p < 0.05; **p < 0.01; ***p < 0.001.

To examine the relationship between deregulated DNM3 expression and LC development, RT-PCR analysis of DNM3 expression in tissue samples obtained from 51 pairs of LC tumors and adjacent tissue from LC patients. The RT-PCR analysis revealed that the DNM3 was lower in the LC tumors at the mRNA level (Figure 1A), although the mRNA levels were higher in LC tissues than in adjacent normal lung tissue for many patients. Among these 51 patients, 23 patients were found metastasis. We then further compared the DNM3 expression level in the tumor from these 23 patients with metastasis and 28 patients without metastasis. The patients with tumor metastasis had lower *DNM3* mRNA levels (Figure 1B), suggesting that the low expression of *DNM3* might also contribute to metastasis of the tumor. DNM3 expression was then evaluated in a non-cancerous pulmonary epithelial cell line (BEAS-2B) and 5 LC cell lines (A549, H460, H1299, Calu-3, and H1838). The mRNA and protein expression levels of DNM3 were significantly lower in the H1838, A549, and H1299 cells than in the BEAS-2B cells (Figures 1C,D). Consistently, the data from the cancer browser^1^ also suggested that DNM3 mRNA expression level is lower in the LC tumors than the solid normal lung tissues (Figure 1E). A Kaplan–Meier plot^2^ analysis revealed that a lower DNM3 level was associated with poor OS of patients with LC (Figure 1F). Thus, our data indicated that DNM3 expression is lower in lung tumors, especially in those with metastasis. Furthermore, the low expression of DNM3 leads to poor clinical outcomes in patients with LC.

### DNM3 Expression Decreases Lung Cancer Cell Proliferation and Migration

**FIGURE 2:**
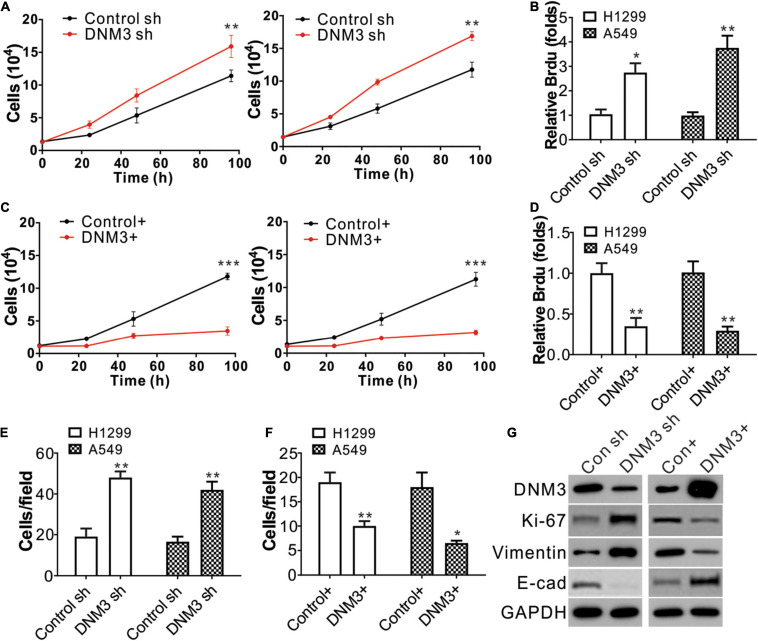
The knockdown of DNM3 promotes lung cancer cell proliferation and migration *in vitro*. **(A)** MTS assay of the growth of H1299 (left) and A549 (right) cells with or without DNM3 shRNA transfection. **(B)** Brdu assay of the proliferation of H1299 and A549 cells with or without DNM3 shRNA transfection. **(C)** MTS assay of the growth of H1299 (left) and A549 (right) cells with or without DNM3 overexpression. **(D)** Brdu assay of the proliferation of H1299 and A549 cells with or without DNM3 overexpression. **(E)** Transwell assay of the migration of H1299 and A549 cells with or without *DNM3* shRNA transfection. **(F)** Transwell assay of the migration of H1299 and A549 cells with or without DNM3 overexpression. **(G)** Expression of the indicated proteins in H1299 cells with *DNM3* shRNA transfection (left) or *DNM3* overexpression (right). Each experiment was repeated 3 times. *p < 0.05; **p < 0.01; ***p < 0.001.

Next, we investigated whether the DNM3 expression level is related to LC progression. Lentivirus transfection was used to deliver shRNA into the A549 and H1299 cell lines to knock down *DNM3*. The depletion of DNM3 in these LC cells significantly promoted cell proliferation compared to that of the control shRNA-transfected cells (Figure 2A). The BrdU assay also indicated that the proliferation of the LC cells was enhanced by the knockdown of *DNM3* (Figure 2B). By contrast, the enhancement of DNM3 expression by pLenti-*DNM3* transfection suppressed H1299 and A549 cell proliferation (Figures 2C,D). Similarly, knowdown of *DNM3* also promotes BEAS-2B cell growth (Supplementary Figures 1A,B), indicating the tumor-suppressive role of DNM3.

Additionally, the effect of DNM3 on the migration of LC cells was investigated. The transwell migration assay revealed that the absence of DNM3 promoted the migration of the A549 and H1299 cells (Figure 2E and Supplementary Figure 1C), whereas the overexpression of DNM3 had the opposite effect (Figure 2F and Supplementary Figure 1D). To further confirm the role of DNM3 in LC proliferation and metastasis, the expression levels of a proliferation marker (Ki-67) (Scholzen and Gerdes, 2000) and migration marker (E-cadherin, Vimentin) (Lee et al., 2006) were analyzed by western blot assay. Consistently, the knockdown of *DNM3* in H1299 cells enhanced the level of Ki-67 and Vimentin, and decreased the level of E-cadherin (Figure 2G). This scenario was reversed by *DNM3* overexpression (Figure 2G). Therefore, these results indicate that DNM3 expression is correlated with the proliferation and migration of LC cells.

### Knockdown of *DNM3* Enhances Lung Cancer Cell Proliferation and Metastasis *in vivo*

**FIGURE 3:**
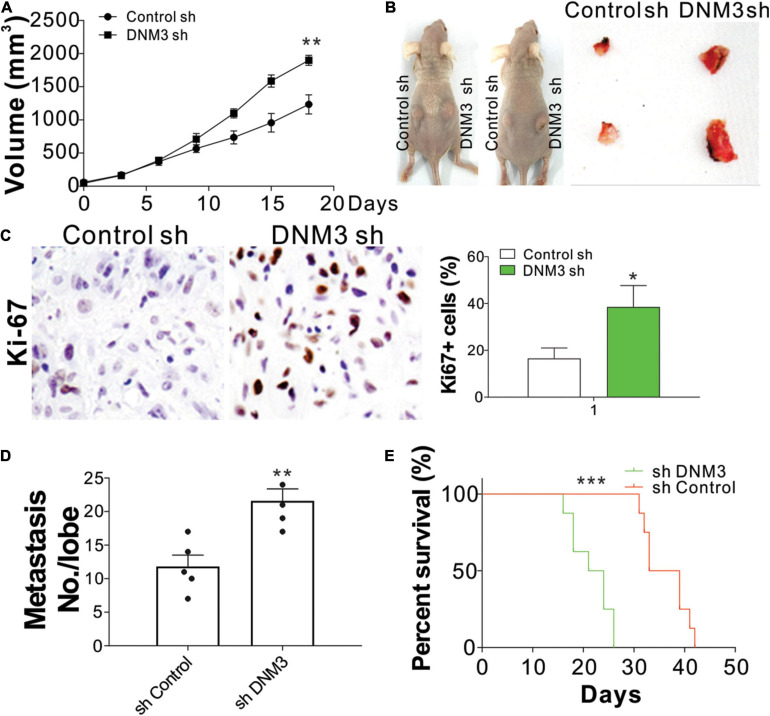
Reduced DNM3 expression promotes lung cancer tumor growth and metastasis *in vivo*. **(A)** Tumor growth in nude mice xenografted with H1299 cells with or without stable *DNM3* knockdown (*n* = 5 for each group). **(B)** Representative picture of xenografted tumors. **(C)** Ki-67 staining of tumors in each group. **(D)** Metastatic tumors in the lung tissue of nude mice (*n* = 5 for each group) injected through the tail vein with H1299 cells with or without stable *DNM3* knockdown. **(E)** Survival of nude mice (*n* = 8 for each group) injected through the tail vein with H1299 cells with or without stable *DNM3* knockdown. Experiment in C was repeated 3 times. *p < 0.05; **p < 0.01; ***p < 0.001.

To confirm the effect of DNM3 on LC growth *in vivo*, H1299 xenografts in nude mice were examined. The mice were subcutaneously inoculated with H1299 cells with or without stable *DNM3* knockdown. The tumors with DNM3 knockdown had a faster growth rate and larger tumor size, when compared with the tumors with control shRNA transfection (Figures 3A,B). Furthermore, immunochemistry staining revealed that depletion of DNM3 enhanced the Ki-67 expression in the LC xenografted tumors (Figure 3C). To examine the absence of DNM3 for the promotion of metastasis, a tail vein tumor metastasis model was used. H1299 cells with or without stable *DNM3* knockdown were injected into nude mice through the tail vein injection. Three weeks later, the mice were sacrificed for the observation of lung tumor formation. Hematoxylin-eosin staining was used to observe the metastatic tumors in the lung tissue sections. It was found that the mice injected with *DNM3*-shRNA transfected H1299 cells had a higher number of metastatic tumors (Figure 3D). Without DNM3 in the tumor cells, the mice had a worse survival rate (Figure 3E). These results confirmed the tumor-suppressive role of DNM3 in LC *in vivo*.

### The Absence of DNM3 Promotes Lung Cancer Proliferation and Migration Through STAT3 Activation

**FIGURE 4:**
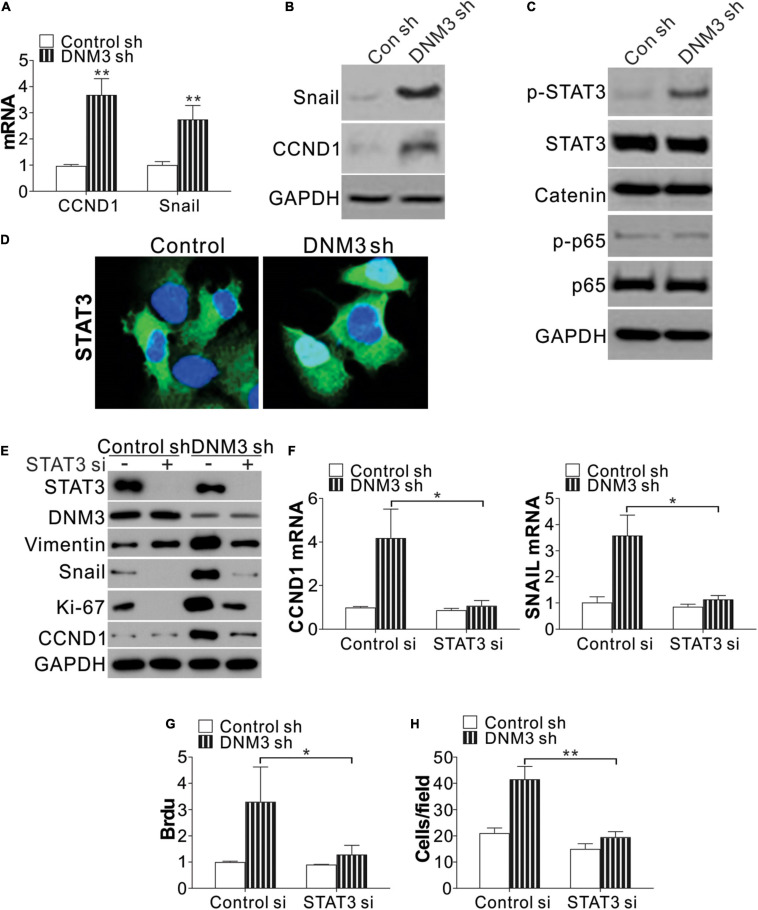
Silencing of DNM3 leads to STAT3 activation. **(A)** mRNA levels of *CCND1* and *SNAI1* in H1299 cells with or without *DNM3* shRNA transfection. **(B)** Protein levels of CCND1 and SNAI1 in H1299 cells with or without *DNM3* shRNA transfection. **(C)** Protein level of p-STAT3, STAT3, beta-catenin, p-p65, and p65 in H1299 cells with or without *DNM3* shRNA transfection. **(D)** Immunostaining of STAT3 in H1299 cells with or without *DNM3* shRNA transfection. **(E–H)** H1299 cells with or without stable DNM3 knockdown were transfected with control or *STAT3* siRNA. **(E)** Western blot assay of the indicated proteins. **(F)** RT-qPCR assay of the mRNA expression of *CCND1* and *SNAI1*. GAPDH was used as reference gene. **(G)** Brdu assay of cell proliferation. **(H)** Transwell assay of the migration of the cells. Each experiment was repeated 3 times. *p < 0.05; **p < 0.01.

To investigate the function of DNM3 in LC proliferation and migration, we analyzed the expression of two genes related to the proliferative and metastatic capacities of the cells, *CCDN1* and *SNAI1*. The mRNA expression of these two genes was found to be significantly upregulated in the *DNM3*-knockdown H1299 cells (Figure 4A). Consistently, the protein levels of CCND1 and SNAI1 were also increased in these DNM3-depleted cells (Figure 4B). Since the increased expression of *CCND1* and *SNAI1* was mediated by *DNM3* knockdown at the mRNA level, we then analyzed the expression of their transcription factors, including STAT3, beta-catenin, and nuclear factor-kappa B (NF-κB) (p65). It was found that only STAT3 phosphorylation was upregulated in the *DNM3*-depleted H1299 cells (Figure 4C). Immunostaining of STAT3 revealed that its nuclear translocation was promoted in the absence of DNM3 (Figure 4D), further indicating the activation of STAT3 upon DNM3 depletion. To verify the role of STAT3 in the *DNM3*-depletion-mediated promotion of CCND1 and SNAI1 expression, *STAT3* was silenced by siRNA transfection in *DNM3*-knockdown H1299 cells. As a result, the upregulation of the CCND1 and SNAI1 protein and mRNA levels in these cells was abolished (Figures 4E,F). The silencing of *STAT3* also suppressed the cell proliferation and migration induced by DNM3 depletion in the H1299 cells (Figures 4E,G,H). Therefore, our results collectively indicated that STAT3 activation promoted the LC proliferation and migration induced by DNM3 depletion.

### DNM3 Forms a Complex With GRB2 to Suppress STAT3 Activation

**FIGURE 5:**
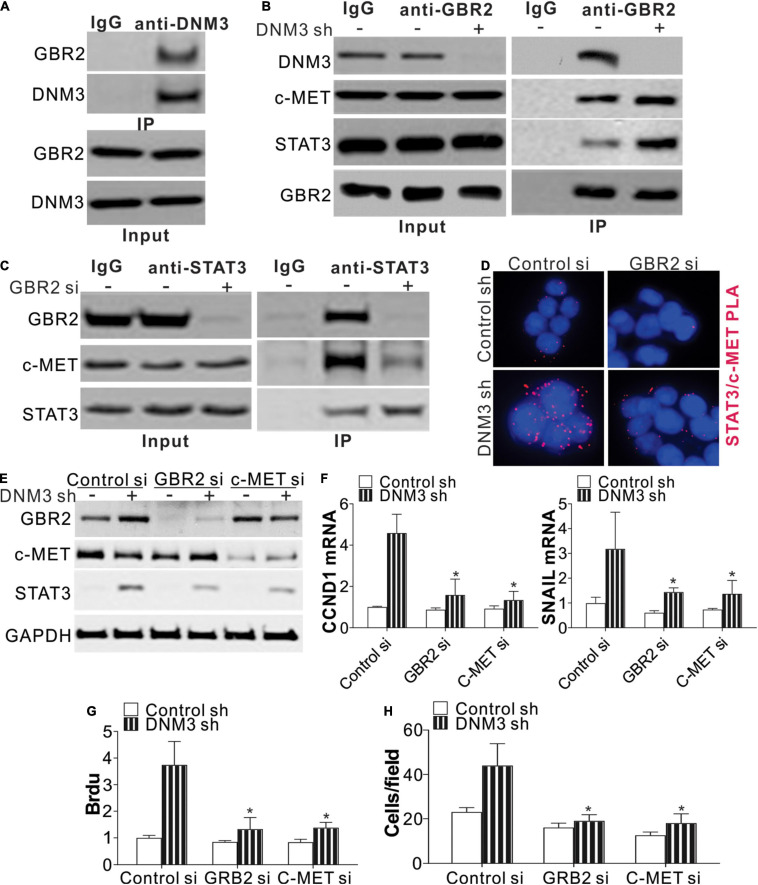
DNM3 interacts with GBR2 to suppress the latter’s binding with STAT3 and c-MET. **(A)** Immunoprecipitation assay of the interaction between DNM3 and GBR2, using anti-DNM3 antibody. **(B)** Interaction among GBR2, STAT3, c-MET, and DNM3 in H1299 cells with or without *DNM3* knockdown. **(C)** Interaction of STAT3 and c-MET with GBR2 in H1299 cells transfected with control or *GBR2* siRNA. **(D)** H1299 cells with or without stable *DNM3* knockdown were transfected with control or *GBR2* siRNA. The interaction of STAT3 with c-MET was analyzed by proximity ligation assay. **(E–H)** H1299 cells with or without stable *DNM3* knockdown were transfected with control, *GBR2*, or *c-MET* siRNA. **(E)** Western blot assay of the indicated proteins. **(F)** RT-qPCR assay of the mRNA expression of *CCND1* and *SNAI1*. GAPDH was used as reference gene. **(G)** Brdu assay of cell proliferation. **(H)** Transwell assay of the migration of the cells. Each experiment was repeated 3 times. *p < 0.05.

Next, we studied the mechanism of STAT3 activation in the absence of DNM3. A protein-protein interaction tool predicted that DNM3 could interact with GBR2 (Brehme et al., 2009), which is necessary for STAT3 activation by c-MET. Using immunoprecipitation, we found that DNM3 is bound directly with GBR2 (Figure 5A). Under the DNM3 depletion condition, the interaction of GBR2 with c-MET and STAT3 was enhanced (Figure 5B), further indicating that DNM3 competes with STAT3 and c-MET to bind with GBR2. Furthermore, the absence of GBR2 reduced the interaction between STAT3 and c-MET (Figure 5C). These results indicated that DNM3 suppressed STAT3 activation through competitive binding with GBR2. To confirm our hypothesis, we analyzed the interaction between c-MET and STAT3 using a proximity ligation assay. Consistently, we found that the absence of DNM3 enhanced the interaction between c-MET and STAT3, and this interaction was abolished by *GBR2*-siRNA knockdown (Figure 5D). Next, we analyzed the roles of GBR2 and c-MET in STAT3 activation without DNM3. The knockdown of either GBR2 or c-MET by their respective siRNAs compromised the phosphorylation of STAT3 (Figure 5E) and inhibited CCND1 and SNAI1 expression at the protein and mRNA levels (Figures 5E,F). Consistently, the absence of GBR2 or c-MET also suppressed the LC cell growth and cell migration induced by DNM3 depletion (Figures 5G,H). Collectively, our data suggested that DNM3 suppresses LC tumor growth and migration by interacting with GBR2, which subsequently leads to the dissociation of the c-MET and STAT3 complex.

### c-MET Inhibition Suppresses Lung Cancer Progression With DNM3 Silenced

**FIGURE 6:**
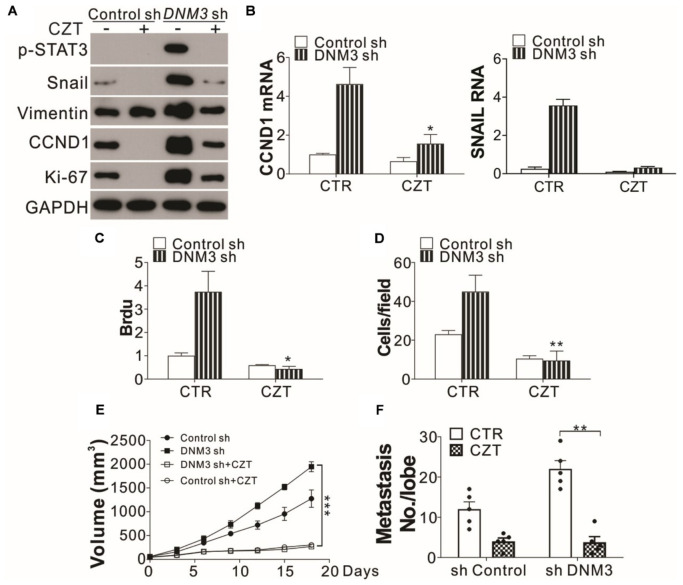
The c-MET inhibitor has tumor suppressive effects on DNM3-depleted lung cancer cells and tumors. **(A–D)** H1299 cells with or without stable *DNM3* knockdown were treated with 10 μM crizotinib (CZT) for 24 h. **(A)** Western blot assay of the expression of the indicated proteins. **(B)** RT-qPCR assay of the mRNA levels of *CCND1* and *SNAI1*. **(C)** Brdu assay of cell proliferation. **(D)** Transwell assay of the migration of the cells. **(E)** Nude mice xenografted with H1299 cells with or without stable *DNM3* knockdown were treated with CZT (35 mg/kg per day for 12 days) by oral gavage (*n* = 5 for each group). The tumor growth was monitored. **(F)** Nude mice (*n* = 5 for each group) injected via the tail vein with H1299 cells with or without stable *DNM3* knockdown were treated with CZT (35 mg/kg per day for 12 days) by oral gavage (*n* = 5 for each group). The number of metastatic tumor nodules in the lung was counted. Experiments for **(A–D)** were repeated 3 times. *p < 0.05; **p < 0.01; ***p < 0.001.

Since the knockdown of c-MET suppressed LC tumor growth and cell migration under a DNM3 depletion condition, the c-MET inhibitor CZT was investigated for its antitumor potential. Similar to the effect of c-MET knockdown, CZT treatment suppressed the activation of STAT3 and the mRNA and protein induction of SNAIl and CCND1 in the DNM3-depleted cells (Figures 6A,B). CZT also suppressed the proliferation and migration of both H1299 cells with and without *DNM3* knockdown (Figures 6A,C,D). Next, we investigated the *in vivo* effect of CZT in suppressing the progression of xenografted H1299 cell-derived tumors (with or without *DNM3* knockdown) in nude mice. We found CZT treatment in nude mice completely compromised the tumor growth of DNM3 stably knockdown H1299 cells xenografts (Figure 6E). And the inhibitory effect of CZT was similar in the tumors xenografted with H1299 cells transfected with control shRNA (Figure 6E). In terms of cancer metastasis, our results showed that CZT treatment also reduced the tumor metastasis regardless of the status of DNM3 (Figure 6F). The CZT treatment did not affect the body weight of mice in both the xenograft model and tail vein metastasis model (Supplementary Figures 2A,B). Therefore, our data suggested that low DNM3 expression leads to c-MET activation, and c-MET inhibitors could be used to treat LC tumors with low DNM3 expression.

## Discussion

This study showed that DNM3 was downregulated in LC tissue cells and that the downregulation of this enzyme promoted the proliferative and metastatic capacities of the cells by increasing CCND1 and SNAI1 expression. The *in vivo* experiments showed that the lack of DNM3 could improve the proliferative and metastatic capacities of human LC xenografts in nude mice. Additionally, the mechanism by which DNM3 induced the suppression of LC cell proliferation was through its interaction with GBR2, which disrupted the c-MET–GBR2–STAT3 complex formation. The administration of the c-MET inhibitor CZT to the mice could overcome the LC proliferation and metastasis induced by the DNM3 depletion.

DNM3, which belongs to the dynamin family, is a GTPase essential in endocytosis and possessing mechanochemical properties of tabulating and severing membranes (Inokawa et al., 2013). The association between DNM3 and cancer was unknown until Shen et al. (2012) first reported the hypermethylation of *DNM3* in HCC tissues, which indicated that it might act as a cancer suppressor. Inokawa et al. (2013) subsequently showed that the methylation of the *DNM3* promoter served to downregulate its expression and was related to the poor prognosis of patients with HCC, thus suggesting that *DNM3* may be a novel tumor suppressor gene. Furthermore, Lin et al. (2019) found that the DNM3 level in a papillary thyroid cancer cell line was significantly reduced. Although the function of DNM3 in other tumor types has been rarely reported, the findings from the studies mentioned above suggested that this enzyme may also have an anti-cancer function in LC, with a similar expression pattern to that in HCC. It was reported that DNM3 could hinder mitosis by inducing G_0_/G_1_ cell cycle arrest (Gu et al., 2017). Similarly, we found that the knockdown of *DNM3* enhanced the expression of CCND1, which is required for progression through the G1 phase of the cell cycle (Baldin et al., 1993). Other than CCND1, the cell migration modulator SNAI1 was also upregulated by DNM3 depletion. The enhancement of CCND1 and SNAI1 expressions was mediated by STAT3, which was overactivated by the DNM3 knockdown, thereby contributing to LC growth and metastasis.

As to the mechanism of STAT3 activation by DNM3 knockdown, DNM3 was found to compete in binding with GRB2, which blocks the formation of the c-MET–GRB2–STAT3 complex and prevents the activation of STAT3. Although STAT3 has also been implicated in cellular transformation, its proposed mechanism is controversial. Constitutive activation of STAT3 is a common feature in LC, and has also been proposed to play an important role in tumor resistance to conventional and targeted small-molecule therapies (Haura et al., 2005; Gao et al., 2007). Generally, the life span of an activated STAT protein is very short as it can be rapidly dephosphorylated by protein phosphatases. However, in various primary tumors and tumor cell lines, STAT3 and STAT5 remain constitutively active (Haura et al., 2005; Gao et al., 2007; Yu et al., 2009). Such sustained STAT activation is due to the increased expression of receptors and kinases (autocrine and paracrine pathways) or decreased activity of suppressors of cytokine signaling (SOCS), protein tyrosine phosphatases (SHP-1 and SHP- 2), protein inhibitor of activated STAT (PIAS), and other negative regulators (Levy and Darnell, 2002; Yu et al., 2009). Herein, we revealed a novel mechanism of persistent STAT3 activation that was associated with low DNM3 expression in LC. The interaction of STAT3 with GBR2 is important for the palmitoylation of STAT3, which results in its activation and translocation to the nucleus (Niu et al., 2019). Although the binding of c-MET to STAT3 was reported to activate the STAT3 phosphorylation directly, GBR2 is an essential mediator for this process (Hass et al., 2017). Our study revealed a novel function of DNM3, in that it could interact with GBR2 and dissociate it from the c-MET–GBR2–STAT3 complex. These data might explain the persistent STAT activation in some cancer types.

Finally, we revealed that a c-MET inhibitor (CZT) could inhibit the cancer cells’ proliferative and metastatic capacities induced by the DNM3 depletion. In NSCLC, dysregulation of the c-MET signal-mediated growth, death, and migration of the cells could be achieved through c-MET overexpression, amplification, mutation, or hepatocyte growth factor−mediated NSCLC activation (Smyth et al., 2014). The efficacy of c-MET inhibitors in patients with large *MET* gene copy numbers has been reported, and this treatment method is being tested in prospective clinical trials (Ou et al., 2011; Salgia, 2017). However, c*-MET* amplification is found in only 5% of patients with newly diagnosed lung adenocarcinoma (Cappuzzo et al., 2009). c-MET suppressors can take effect only if the c-*MET* copy number is more than 5 (Salgia, 2017). Therefore, targeting c-MET in cancer is hampered by a lack of diagnostics that accurately reflect high c-MET signaling and dependence. It was demonstrated that c-MET-GRB2 protein complex abundance predicts c-MET survival signaling and correlates with sensitivity to c-MET inhibitors in c-MET-driven cellular models of lung cancer (Smith et al., 2017). However, the mechanism behind the high formation of c-MET–GRB2 complexes in LC is not clear. Our study has provided a novel mechanism of c-MET–GRB2 complex formation, which DNM3 modulates. Our *in vivo* data showed that c-MET inhibitor (CZT) dramatically suppressed the growth and metastasis of tumors with lower DNM3 expression (Figures 6E,F). And the tumor-suppressive effects were similar to those tumors with normal DNM3 expression (Figures 6E,F). Since DNM3 is frequently expressed at low level in LC, it might be a potential predictor of high c-MET-GRB2 complex formation and clinical usage of c-MET inhibitor.

In conclusion, our data have confirmed the tumor suppressor function of DNM3 in LC and that it is mediated through suppression of the STAT3 signaling pathway. We have also revealed a novel mechanism of preventing STAT3 activation by DNM3 through its competitive binding with GRB2. This novel function of DNM3 means that its expression level could be used as a new biomarker of the therapeutic effect of c-MET inhibitors in patients with LC.

## Data Availability Statement

The original contributions presented in the study are included in the article/Supplementary Material, further inquiries can be directed to the corresponding author/s.

## Ethics Statement

The studies involving human participants were reviewed and approved by The Air Force Military Medical University. The patients/participants provided their written informed consent to participate in this study. The animal study was reviewed and approved by The Air Force Military Medical University.

## Author Contributions

QL, YN, and LS designed the experiments. WW and LW carried out the experiments and analyzed the experimental results. QL and YN wrote the manuscript. TJ and LS revised the manuscript. All authors approved the final manuscript.

## Conflict of Interest

The authors declare that the research was conducted in the absence of any commercial or financial relationships that could be construed as a potential conflict of interest.

## Publisher’s Note

All claims expressed in this article are solely those of the authors and do not necessarily represent those of their affiliated organizations, or those of the publisher, the editors and the reviewers. Any product that may be evaluated in this article, or claim that may be made by its manufacturer, is not guaranteed or endorsed by the publisher.

## Footnotes

1
https://xena.ucsc.edu/welcome-to-ucsc-xena/

2
http://kmplot.com/analysis/index.php?p=service&cancer=lung

## Supplementary Material

Supplementary Figure 1 Low expression DNM3 promotes cell proliferation and migration *in vitro*. **(A)** MTS assay of the growth of BEAS-2B cells with or without DNM3 shRNA transfection. **(B)** Brdu assay of the proliferation of BEAS-2B cells with or without DNM3 shRNA transfection. **(A)** The presentative pictures of transwell assay of the migration of H1299 and A549 cells with or without *DNM3* shRNA transfection. **(F)** The presentative pictures of transwell assay of the migration of H1299 and A549 cells with or without DNM3 overexpression. Each experiment was repeated 3 times. ^∗^*p* < 0.05; ^∗∗^*p* < 0.01.

Supplementary Figure 2 The administration of crizotinib (CZT) did not affect the body weight of mice. **(A)** Nude mice xenografted with H1299 cells with or without stable *DNM3* knockdown were treated with CZT (35 mg/kg per day for 12 days) by oral gavage (*n* = 5 for each group). The body weight of mice was monitored. **(B)** Nude mice (*n* = 5 for each group) injected via the tail vein with H1299 cells with or without stable *DNM3* knockdown were treated with CZT (35 mg/kg per day for 12 days) by oral gavage (*n* = 5 for each group). The body weight of mice was monitored. NS, *p* > 0.05.
