# Corrigendum: Dynamin 3 inhibits the proliferation of non-small-cell lung cancer cells by suppressing c-MET–GBR2–STAT3 complex formation

**DOI:** 10.3389/fcell.2025.1566930

**Published:** 2025-05-13

**Authors:** Qiang Lu, Yunfeng Ni, Wuping Wang, Lei Wang, Tao Jiang, Lei Shang

**Affiliations:** ^1^ Department of Thoracic Surgery, Tangdu Hospital, The Air Force Military Medical University, Xi’an, China; ^2^ The Ministry of Education Key Lab of Hazard Assessment and Control in Special Operational Environment, Department of Health Statistics, School of Public Health, The Air Force Military Medical University, Xi’an, China

**Keywords:** dynamin 3, lung cancer, non-small-cell lung cancer, growth factor receptor-bound protein 2, antitumor effect

In the published article, there was an error in Figure 6B as published. The wrong bar chart for SNAIL mRNA was erroneously inserted for Figure 6B. The corrected Figure 6 and its caption appear below.

**FIGURE 6:**
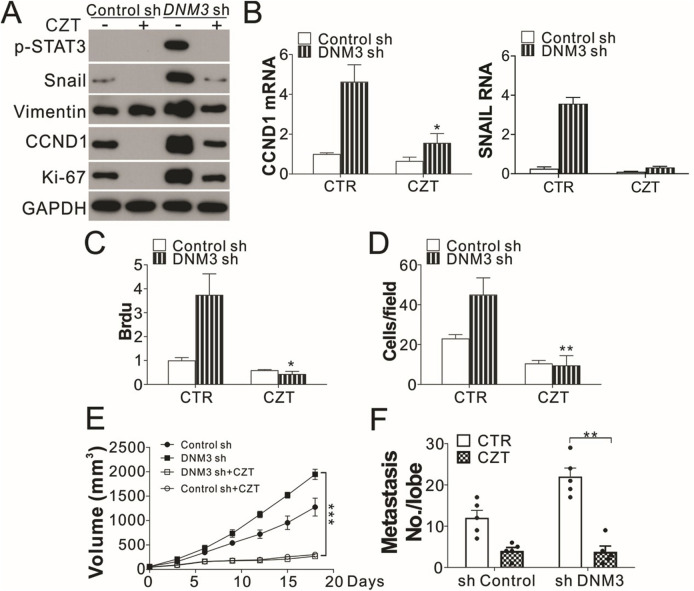
The c-MET inhibitor has tumor suppressive effects on DNM3-depleted lung cancer cells and tumors. **(A–D)** H1299 cells with or without stable DNM3 knockdown were treated with 10 µM crizotinib (CZT) for 24 h. **(A)** Western blot assay of the expression of the indicated proteins. **(B)** RT-qPCR assay of the mRNA levels of CCND1 and SNAI1. **(C)** Brdu assay of cell proliferation. **(D)** Transwell assay of the migration of the cells. **(E)** Nude mice xenografted with H1299 cells with or without stable DNM3 knockdown were treated with CZT (35 mg/kg per day for 12 days) by oral gavage (n = 5 for each group). The tumor growth was monitored. **(F)** Nude mice (n = 5 for each group) injected via the tail vein with H1299 cells with or without stable DNM3 knockdown were treated with CZT (35 mg/kg per day for 12 days) by oral gavage (n = 5 for each group). The number of metastatic tumor nodules in the lung was counted. Experiments for **(A–D)** were repeated 3 times. *p < 0.05; **p < 0.01; ***p < 0.001.

The authors apologize for this error and state that this does not change the scientific conclusions of the article in any way. The original article has been updated.

