# Supplementary figures and images for: Dynamin 3 Inhibits the Proliferation of Non-small-Cell Lung Cancer Cells by Suppressing c-MET–GBR2–STAT3 Complex Formation
Source: Front Cell Dev Biol. 2021 Aug 19;9:641403. doi: 10.3389/fcell.2021.641403 (PMC8416685; doi:10.3389/fcell.2021.641403)

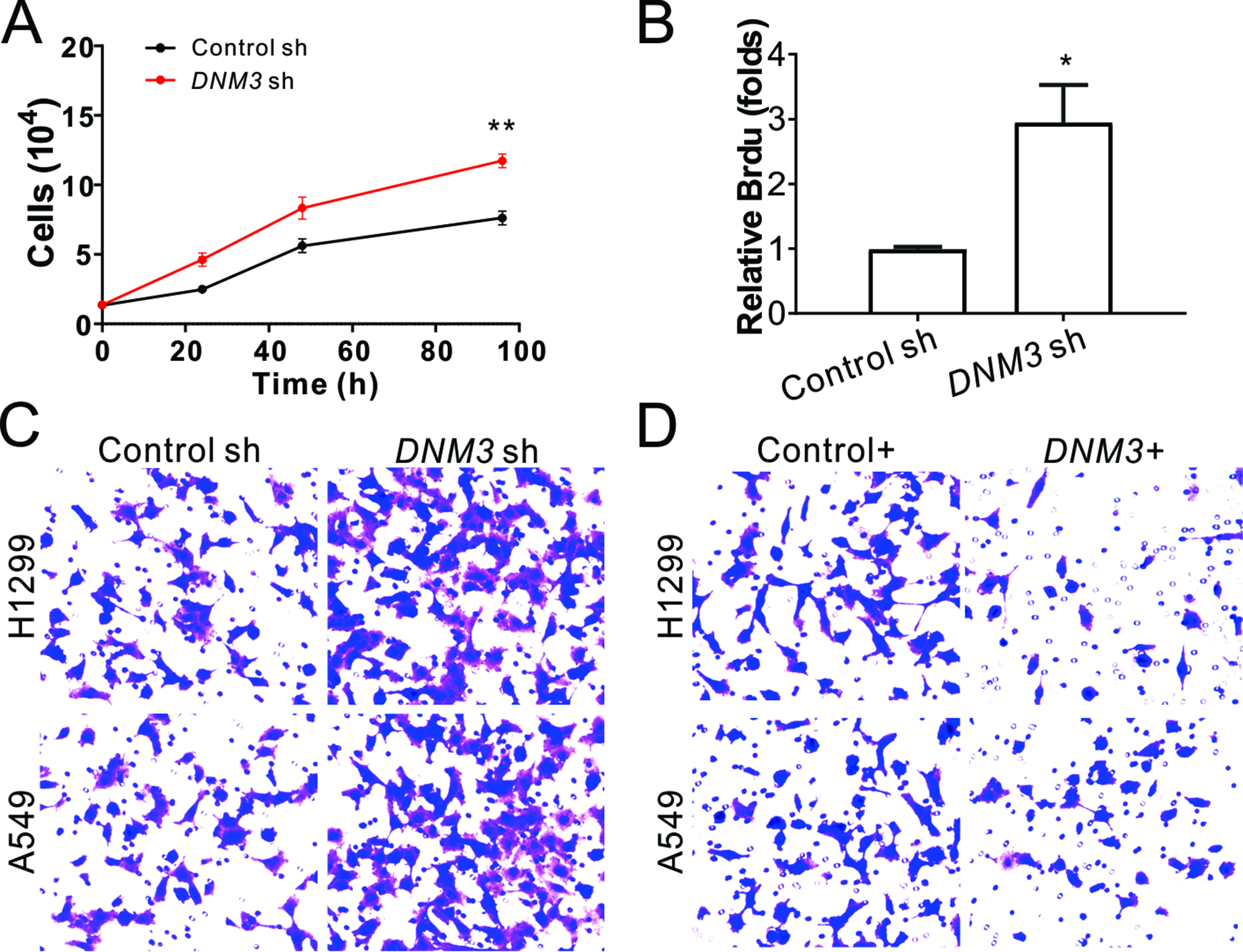

Supplement: Supplementary Figure 1 — Low expression DNM3 promotes cell proliferation and migration in vitro. (A) MTS assay of the growth of BEAS-2B cells with or without DNM3 shRNA transfection. (B) Brdu assay of the proliferation of BEAS-2B cells with or without DNM3 shRNA transfection. (A) The presentative pictures of transwell assay of the migration of H1299 and A549 cells with or without DNM3 shRNA transfection. (F) The presentative pictures of transwell assay of the migration of H1299 and A549 cells with or without DNM3 overexpression. Each experiment was repeated 3 times. ∗p < 0.05; ∗∗p < 0.01. [file Image_1.TIF]

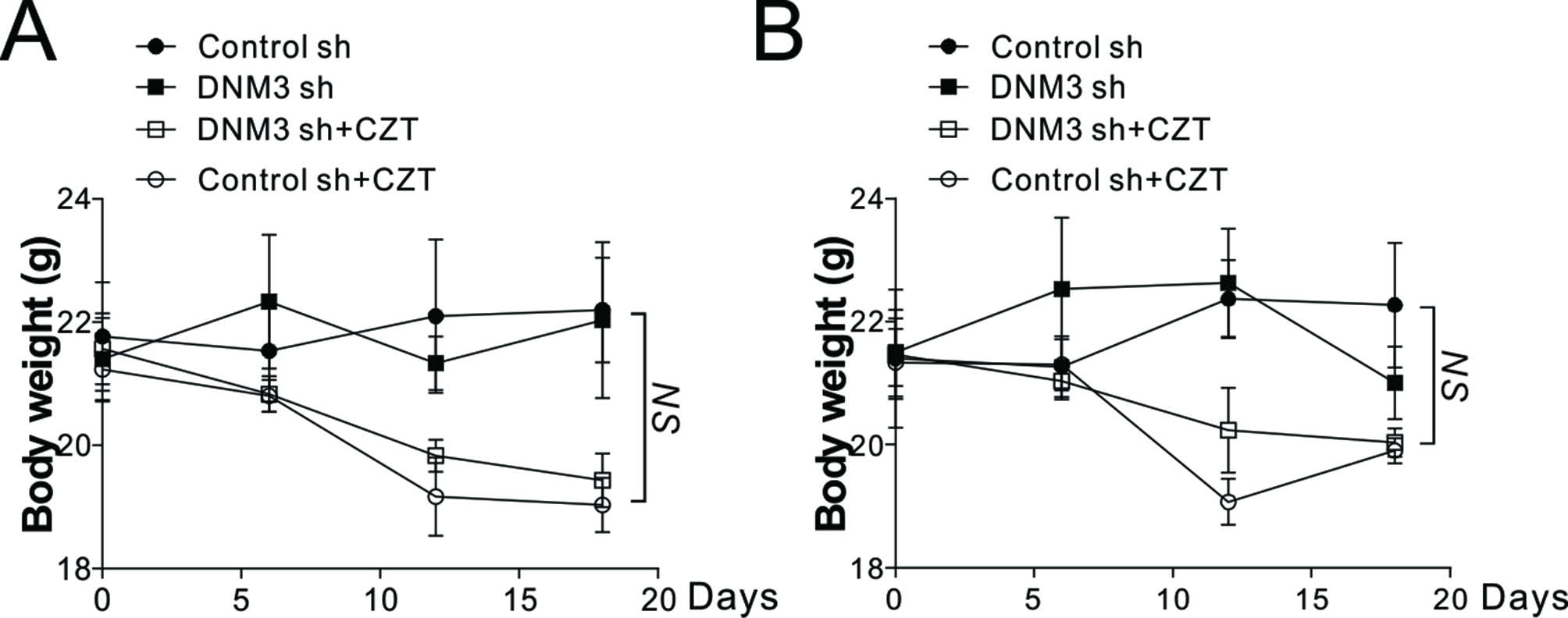

Supplement: Supplementary Figure 2 — The administration of crizotinib (CZT) did not affect the body weight of mice. (A) Nude mice xenografted with H1299 cells with or without stable DNM3 knockdown were treated with CZT (35 mg/kg per day for 12 days) by oral gavage (n = 5 for each group). The body weight of mice was monitored. (B) Nude mice (n = 5 for each group) injected via the tail vein with H1299 cells with or without stable DNM3 knockdown were treated with CZT (35 mg/kg per day for 12 days) by oral gavage (n = 5 for each group). The body weight of mice was monitored. NS, p > 0.05. [file Image_2.TIF]
